# ﻿Two new millipede species of the genus *Coxobolellus* Pimvichai, Enghoff, Panha & Backeljau, 2020 (Diplopoda, Spirobolida, Pseudospirobolellidae)

**DOI:** 10.3897/zookeys.1128.94242

**Published:** 2022-11-09

**Authors:** Piyatida Pimvichai, Henrik Enghoff, Thierry Backeljau

**Affiliations:** 1 Department of Biology, Faculty of Science, Mahasarakham University, Maha Sarakham 44150, Thailand Mahasarakham University Maha Sarakham Thailand; 2 Natural History Museum of Denmark, University of Copenhagen, Universitetsparken 15, DK-2100 Copenhagen Ø, Denmark University of Copenhagen Copenhagen Denmark; 3 Royal Belgian Institute of Natural Sciences, Vautierstraat 29, B-1000 Brussels, Belgium Royal Belgian Institute of Natural Sciences Brussels Belgium; 4 Evolutionary Ecology Group, University of Antwerp, Universiteitsplein 1, B-2610 Antwerp, Belgium University of Antwerp Antwerp Belgium

**Keywords:** DNA barcode, gonopod morphology, identification key, taxonomy, Thailand

## Abstract

Two new millipede species of the genus *Coxobolellus* Pimvichai, Enghoff, Panha & Backeljau, 2020 from Thailand are described: *Coxobolellussaratani***sp. nov.** from Loei Province and *Coxobolellusserratoligulatus***sp. nov.** from Uttaradit Province. The descriptions are based on gonopod morphology and two mitochondrial gene fragments (COI and 16S rRNA). The phylogenetic mtDNA analysis assigned the two new species unequivocally to the consistently well-supported *Coxobolellus* clade, in which they form a fifth subclade that was well supported by maximum likelihood analysis of 16S rRNA, though neither by Bayesian inference nor by COI. The two new *Coxobolellus* species share four conspicuous gonopodal synapomorphies of the genus: (1) the protruding process on the coxae of the 3^rd^ (and sometimes 4^th^) pair of male legs, (2) a large, triangular coxae on the 4^th^–5^th^ pair of legs, (3) a short process of the preanal ring protruding as far as, or slightly beyond, the anal valves, and (4) the posterior gonopod telopodite divided into two parts, with a conspicuous pore opening at the mesal margin at the end of the coxal part of the posterior gonopod. Thus, the two new species provide further evidence of the well-defined monophyly of the genus *Coxobolellus*. Finally, the paper provides an updated morphological identification key to all currently described *Coxobolellus* species.

## ﻿Introduction

Until recently, the poorly known millipede family Pseudospirobolellidae Brölemann, 1913 included only four species in two genera, viz. *Pseudospirobolellus* Carl, 1912 and *Benoitolus* Mauriès, 1980 (Enghoff et al. 2015). Yet, the past few years fieldwork in Thailand has led to the discovery of 13 new pseudospirobolellid species and their assignment to two new genera, viz. *Coxobolellus* Pimvichai, Enghoff, Panha & Backeljau, 2020 (10 species) and *Siliquobolellus* Pimvichai, Enghoff, Panha & Backeljau, 2022 (3 species) (Pimvichai et al. 2020, 2022b). As such, the genus *Coxobolellus*, with its unique synapomorphic protruding process on the coxae of the 3^rd^ (and sometimes 4^th^) pair of male legs, appears to be a particularly well supported and species-rich clade (Pimvichai et al. 2020). It was therefore expected that additional *Coxobolellus* species were bound to be discovered. The present study complies with this expectation as it combines morphological and mtDNA sequence data to describe two new species of the genus *Coxobolellus* from Thailand.

## ﻿Material and methods

Live specimens were hand-collected. They were partly preserved in 70% ethanol for morphological study and partly placed in a freezer at –20 °C for DNA analysis.

This research was conducted under the approval of the Animal Care and Use regulations (numbers U1-07304-2560 and IACUC-MSU-037/2019) of the Thai government.

### ﻿Morphology

Gonopods were photographed with a digital microscope camera (Zeiss Stemi 305). Samples for scanning electron microscopy (SEM: Hitachi TM4000Plus) were air-dried directly from alcohol and sputter-coated for 60 s with gold (Hitachi: MC1000). Scanning electron micrographs were taken at the Central Lab of Mahasarakham University. Drawings were made using a stereomicroscope and photographs. Voucher specimens were deposited in the collections of the Museum of Zoology, Chulalongkorn University, Bangkok, Thailand (CUMZ).

### ﻿DNA extraction, amplification and sequencing

Total genomic DNA was extracted from legs of specimens of *Coxobolellussaratani* sp. nov. (CUMZ-D00153 and CUMZ-D00153) from Loei Province and *Coxobolellusserratoligulatus* sp. nov. (CUMZ-D00154-1 and CUMZ-D00154) from Uttaradit Province, Thailand using the PureDireX column based genomic DNA extraction kit (tissue) (Bio-Helix) following the manufacturer’s instructions. PCR amplifications and sequencing of the standard mitochondrial COI and 16S rRNA gene fragments were done as described by Pimvichai et al. (2020). The COI and 16S rRNA gene fragments were amplified with the primers LCO-1490 and HCO-2198 (Folmer et al. 1994) for COI and 16Sar and 16Sbr (Kessing et al. 2004) for 16S rRNA. All new nucleotide sequences have been deposited in GenBank under accession numbers OP580097–OP580100 and OP580512–OP580515 for the partial COI and 16S rRNA fragment sequences respectively. Sample data and voucher codes are provided in Table 1.

**Table 1.** Specimens from which the COI and/or 16S rRNA gene fragments were sequenced. CUMZ, Museum of Zoology, Chulalongkorn University, Bangkok, Thailand; NHMD, Natural History Museum of Denmark; NHMW, Naturhistorisches Museum, Vienna, Austria; NHM, The Natural History Museum, London, United Kingdom. Names of countries are in capitals. Abbreviations after species names refer to the isolate of each sequence. GenBank accession numbers are indicated for each species. — means no sequences were obtained.

	Voucher code	Locality	COI	16S rRNA
**Genus *Apeuthes***				
*A.maculatus* Amc	NHMW-Inv. No.2395	South Annam, Vietnam	MF187404	MF187360
*A.maculatus* Am26	NHMD-621697	Nha Trang, Bao Dai Villas Hotel, in garden, Vietnam	MZ567159	MZ568653
*A.fimbriatus* BMP	CUMZ-D00144	Bach Ma Peak, Da Nang, Vietnam	MZ567160	MZ568654
*A.longeligulatus* TPP	CUMZ-D00140	Tham Phet Po Thong, Klong Hard, Sa Kaeo, Thailand	MZ567161	MZ568655
*A.pollex* SMR	CUMZ-D00141	Sra Morakot, Klongthom, Krabi, Thailand	MZ567162	MZ568656
*A.pollex* SML	CUMZ-D00142	Koh 8, Similan islands, Phang-Nga, Thailand	MZ567163	MZ568657
*A.pollex* WTS	CUMZ-D00143	Tham Sue Temple, Muang, Krabi, Thailand	MZ567164	MZ568658
?*A.spininavis* ABB	CUMZ-D00145	Air Banun, Perak, Malaysia	MZ567165	MZ568659
**Genus *Atopochetus***				
*A.anaticeps* SVL	CUMZ-D00091	Srivilai temple, Chalermprakiet, Saraburi, Thailand	MF187405	—
*A.dollfusii* DOL	NHM	Cochinchina, Vietnam	MF187412	MF187367
*A.helix* SPT	CUMZ-D00094	Suan Pa Thong Pha Phum, Kanchanaburi, Thailand	MF187416	MF187371
*A.moulmeinensis* TAK	CUMZ-D00095	Km 87, Tha Song Yang, Tak, Thailand	MF187417	MF187372
*A.setiferus* HPT	CUMZ-D00097	Hub Pa Tard, Lan-Sak, Uthaithani, Thailand	MF187419	MF187374
*A.spinimargo* Ton27	NHMD-00047013	Koh Yo, Songkhla, Thailand	MF187423	MF187377
*A.truncatus* SML	CUMZ-D00101	Koh 8, Similan islands, Phang-Nga, Thailand	MF187424	MF187378
*A.uncinatus* KMR	CUMZ-D00102	Khao Mar Rong, Bangsapan, Prachuapkhirikhan, Thailand	MF187425	MF187379
*A.weseneri* Tos29	NHMD-00047003	Supar Royal Beach Hotel, Khanom, Nakhonsrithammarat, Thailand	MF187431	MF187384
**Genus *Aulacobolus***				
*A.uncopygus* Auc	NHMW-Inv. No.2375	Nilgiris, South India, India	MF187433	MF187386
**Genus *Benoitolus***				
*B.birgitae* BBG	NHMD 621687	Chiang Dao, Chiang-Mai, Thailand	MT328992	—
**Genus *Coxobolellus***				
*C.albiceps* Stpw	CUMZ-D00121	Tham Pha Tub, Muang District, Nan Province, Thailand (green individual)	MT328994	MT328211
*C.albiceps* Stpl	CUMZ-D00122	Tham Pha Tub, Muang District, Nan Province, Thailand (small, brown individual)	MT328993	—
*C.albiceps* TPB	CUMZ-D00123	Wat Tham Bampen Bun, Pan District, Chiang-Rai Province, Thailand	MT328996	MT328213
*C.albiceps* Stvd	CUMZ-D00124	Tham Wang Daeng, Noen Maprang District, Phitsanulok Province, Thailand	MT328995	MT328212
*C.compactogonus* SKR	CUMZ-D00134	Sakaerat Environmental Research Station, Wang Nam Khiao District, Nakhon Ratchasima Province, Thailand	MT328998	MT328215
*C.compactogonus* KLC	CUMZ-D00135	Khao Look Chang, Pak Chong District, Nakhon Ratchasima Province, Thailand	MT328997	MT328214
*C.fuscus* HKK	CUMZ-D00133	Kroeng Krawia waterfall, Sangkhla Buri District, Kanchanaburi Province, Thailand	MT328999	MT328216
*C.nodosus* SPW	CUMZ-D00126	Chao Por Phawo Shrine, Mae Sot District, Tak Province, Thailand	MT329000	MT328217
*C.serratus* KKL	CUMZ-D00132	Khao Kalok, Pran Buri District, Prachuap Khiri Khan Province, Thailand	MT329001	MT328218
*C.simplex* TNP	CUMZ-D00136	Tham Pha Pha Ngam, Mae Prik District, Lampang Province, Thailand	MT329002	—
*C.tenebris* KWP	CUMZ-D00119	Wat Khao Wong Phrohm-majan, Ban Rai District, Uthai Thani Province, Thailand	MT329003	MT328219
*C.tenebris* TPL	CUMZ-D00120	Wat Tham Phrom Lok Khao Yai, Sai Yok District, Kanchanaburi Province, Thailand	MT329004	MT328220
*C.tigris* TKP	CUMZ-D00130	Wat Tham Khao Plu, Pathio District, Chumphon Province, Thailand	MT329005	MT328221
*C.tigris* TYE	CUMZ-D00131	Tham Yai I, Pathio District, Chumphon Province, Thailand	MT329006	MT328222
*C.transversalis* Stpg	CUMZ-D00125	Tham Pha Tub, Muang District, Nan Province, Thailand	MT329007	MT328223
*C.valvatus* TCD	CUMZ-D00127	Wat Tham Chiang Dao, Chiang Dao District, Chiang-Mai Province, Thailand	MT329009	—
*C.valvatus* BRC	CUMZ-D00128	Tham Borichinda, Chom Thong District, Chiang-Mai Province, Thailand	MT329008	MT328224
*C.valvatus* TST	CUMZ-D00129	Tham Sam Ta, Muang District, Mae Hong Son Province, Thailand	MT329010	MT328225
*C.saratani* sp. nov. PPLL2	CUMZ-D00153	Phu Pha Lom, Muang District, Loei Province, Thailand	OP580097	OP580512
*C.saratani* sp. nov. PPLL	CUMZ-D00153-1	Phu Pha Lom, Muang District, Loei Province, Thailand	OP580098	OP580513
*C.serratoligulatus* sp. nov. TCU	CUMZ-D00154	Tham Chan, Thong Saen Khan District, Uttaradit Province, Thailand	OP580099	OP580514
*C.serratoligulatus* sp. nov. TCU2	CUMZ-D00154-1	Tham Chan, Thong Saen Khan District, Uttaradit Province, Thailand	OP580100	OP580515
**Genus *Leptogoniulus***				
*L.sorornus* BTN	CUMZ- D00109	Botanical Garden, Penang, Malaysia	MF187434	MF187387
**Genus *Litostrophus***				
*L.chamaeleon* PPT	CUMZ- D00111	Phu Pha terb, Mukdahan, Thailand	MF187436	MF187389
*L.saraburensis* PKS	CUMZ- D00113	Phukhae Botanical Garden, Saraburi, Thailand	MF187438	MF187391
*L.segregatus* Ls19	NHMD 621686	Koh Kut, Trad, Thailand	MF187440	MF187394
**Genus *Macrurobolus***				
*M.macrurus* INT	CUMZ- D00147	Wat Tham Inthanin, Mae Sot District, Tak Province, Thailand	MZ905519	—
**Genus *Madabolus***				
*M.maximus* Mm4	NHMD-00047007	de Toliara Province, Parc National de Bermaraha, South Bank of Manambolo River, Near Tombeau Vazimba, Madagascar	MF187441	MF187395
**Genus *Narceus***				
*N.annularis*			NC_003343.1	—
**Genus *Parabolus***				
*P.dimorphus* Pd34	NHMD-00047004	Dar es Salaam, Tanzania	MF187442	MF187396
**Genus *Paraspirobolus***				
*P.lucifugus*			AB608779.1	—
**Genus *Pelmatojulus***				
*P.tigrinus* Pt2	NHMD-00047008	Southern part of the Comoé N.P., 30 km north of Kakpin, Côte d’Ivoire	MF187443	MF187397
*P.togoensis* Pto6	NHMD-00047006	Biakpa, Ghana	MF187444	MF187398
**Genus *Pseudospirobolellus***				
*Pseudospirobolellusavernus* GPG	CUMZ-D00117	Gua Pulai, Gua Musang, Kelantan, Malaysia	MT329011	MT328226
*Pseudospirobolellus* sp. KCS	CUMZ-D00118	Koh Chuang, Sattahip, Chonburi, Thailand	MT329012	MT328227
**Genus *Rhinocricus***				
*R.parcus* Rp49	NHMD-00047009	Puerto Rico, USA	MF187449	MF187403
**Genus *Siliquobolellus***				
*S.amicusdraconis*	CUMZ- D00149	Hub Pa Tard, Lan-Sak, Uthaithani, Thailand	OP174621	—
*S.constrictus*	CUMZ- D00150	Ban Yang Chum, Kui Buri, Prachuap Khiri Khan, Thailand	OP174622	—
*S.prasankokae*	CUMZ- D00148	Pha Thai, Ngao, Lampang, Thailand	OP174623	—
**Genus *Trachelomegalus***				
*T.* sp. Tr54	NHMD-00047012	Borneo Sabah, Malaysia	MF187445	—
**Genus *Trigoniulus***				
*T.corallinus* Tco15	NHMD-00047010	Vientiane, Laos	MF187446	MF187400
**Outgroup**				
**Genus *Anurostreptus***				
*A.barthelemyae* Tlb	CUMZ-D00003	Thale-Ban N.P., Khuan-Don, Satun, Thailand	KC519469	KC519543
**Genus *Chonecambala***				
*C.crassicauda* Ttp	CUMZ-D00001	Ton-Tong waterfall, Pua, Nan, Thailand	KC519467	KC519541
**Genus *Thyropygus***				
*T.allevatus* Bb	CUMZ-D00013	BangBan, Ayutthaya, Thailand	KC519479	KC519552

### ﻿Alignment and phylogenetic analysis

The 16S rRNA data included 51 specimens, representing 12 genera and 37 nominal species of ingroup taxa (Table 1). Three species of the order Spirostreptida, viz. *Anurostreptusbarthelemyae* Demange, 1961 (Harpagophoridae), *Chonecambalacrassicauda* Mauriès & Enghoff, 1990 (Pericambalidae) and *Thyropygusallevatus* (Karsch, 1881) (Harpagophoridae) were used as the outgroup. The same ingroup and outgroup taxa were used for COI, with the addition of: (1) *Coxobolellussimplex* Pimvichai, Enghoff, Panha & Backeljau, 2020, (2) *C.albiceps* (Stpl) Pimvichai, Enghoff, Panha & Backeljau, 2020, (3) *C.valvatus* (TCD) Pimvichai, Enghoff, Panha & Backeljau, 2020, (4) *Siliquobolellusamicusdraconis* Pimvichai, Enghoff, Panha & Backeljau, 2022, (5) *S.constrictus* Pimvichai, Enghoff, Panha & Backeljau, 2022, (6) *S.prasankokae* Pimvichai, Enghoff, Panha & Backeljau, 2022, (7) *Paraspiroboluslucifugus* (Gervais, 1836), (8) *Narceusannularis* Rafinesque, 1820, (9) *Trachelomegalus* sp., (10) *Macrurobolusmacrurus* (Pocock, 1893), (11) *Atopochetusanaticeps* Pimvichai, Enghoff, Panha & Backeljau, 2018, and (12) *Benoitolusbirgitae* (Hoffman, 1981). Hence, the COI data set included 63 specimens, representing 18 genera and 47 nominal species of ingroup taxa (Table 1).

CodonCode Aligner (ver. 4.0.4, CodonCode Corporation) was used to assemble the forward and reverse sequences and to check for errors and ambiguities. All sequences were checked with the Basic Local Alignment Search Tool (BLAST) provided by NCBI and compared with reference sequences in GenBank. They were aligned using MUSCLE (ver. 3.6, see http://www.drive5.com/ muscle; Edgar 2004). The COI alignment consisted of 660 bp and that of 16S rRNA consisted of 458 bp (gaps were excluded by complete-deletion). The sequences were checked for ambiguous nucleotide sites, saturation and phylogenetic signal using DAMBE (ver. 5.2.65; see http://www.dambe.bio.uottawa.ca/DAMBE/dambe.aspx; Xia 2018). MEGA (ver. X, see http://www.megasoftware.net; Kumar et al. 2018) was used to (1) check for stop codons, (2) translate COI protein-coding sequences into amino acids, and (3) calculate uncorrected pairwise p-distances among sequences.

Phylogenetic trees were constructed using maximum likelihood (ML) and Bayesian inference (BI) applied to either the COI and 16S rRNA data separately or combined. The shape parameter of the gamma distribution, based on 16 rate categories, was estimated using maximum likelihood analysis. ML trees were inferred with RAxML (ver. 8.2.12, see http://www.phylo.org/index.php/tools/raxmlhpc2_tgb.html; Stamatakis 2014) through the CIPRES Science Gateway (Miller et al. 2010) using a GTR+G substitution model and 1000 bootstrap replicates to assess branch support. BI trees were constructed with MrBayes (ver. 3.2.7a, see http://www.phylo.org/index.php/tools/mrbayes_xsede.html; Huelsenbeck and Ronquist 2001). Substitution models were inferred using jModeltest (ver. 2.1.10, see https://www.github.com/ddarriba/jmodeltest2/releases; Darriba et al. 2012) applying Akaike Information Criterion weights as selection criterion. This yielded as best models GTR+I+G (–lnL = 17117.3442, gamma shape = 0.6820) for the combined dataset, TrN+ I+G (–lnL = 12352.6533, gamma shape = 0.4820) for COI, and TIM3+ I+G (–lnL = 6495.3310, gamma shape = 0.8870) for 16S rRNA.

BI trees were run for 2 million generations for the combined dataset, 3 million generations for the separate 16S rRNA, and 4 million generations for the separate COI datasets (heating parameter: 0.01 for the combined dataset and 16S rRNA, and 0.02 for COI), sampling every 1000 generations. Convergences were confirmed by verifying that the standard deviations of split frequencies were below 0.01. Then the first 1000 trees were discarded as burn-in, so that the final consensus tree was built from the last 3002 trees for the combined dataset, 4502 trees for the 16S rRNA, and 6002 trees for the COI datasets. Branch support was assessed by posterior probabilities.

For ML trees we consider branches with bootstrap values (BV) of ≥ 70% to be well supported (Hillis and Bull 1993) and < 70% as poorly supported. For BI trees, we consider branches with posterior probabilities (PP) of ≥ 0.95 to be well supported (San Mauro and Agorreta 2010) and below as poorly supported.

## ﻿Results

### ﻿Phylogeny

The uncorrected p-distance between the COI sequences (660 bp) ranged from 0.00 to 0.26 (Suppl. material 1). The mean interspecific sequence divergence within *Coxobolellus* was 0.12 (range: 0.06–0.15). The mean intergeneric sequence divergence between *Coxobolellus* and *Pseudospirobolellus* was 0.21 (range: 0.20–0.23), between *Coxobolellus* and *Siliquobolellus* it was 0.17 (range: 0.14–0.20), and between *Coxobolellus* and *Benoitolusbirgitae* it was 0.21 (range: 0.20–0.23).

The uncorrected p-distance between the 16S rRNA (458 bp) sequences ranged from 0.00 to 0.30 (Suppl. material 2). The mean interspecific sequence divergence within *Coxobolellus* was 0.11 (range: 0.05–0.15). The mean intergeneric sequence divergence between *Coxobolellus* and *Pseudospirobolellus* was 0.21 (range: 0.18–0.24). Currently, there are no 16S rRNA sequences for *Siliquobolellus* and *Benoitolus*.

The uncorrected p-distance between the combined COI + 16S rRNA (1118 bp) sequences ranged from 0.00 to 0.27 (Suppl. material 3). The mean interspecific sequence divergence within *Coxobolellus* was 0.11 (range: 0.05–0.14). The mean intergeneric sequence divergence between *Coxobolellus* and *Pseudospirobolellus* was 0.21 (range: 0.19–0.23).

**Figure 1.**
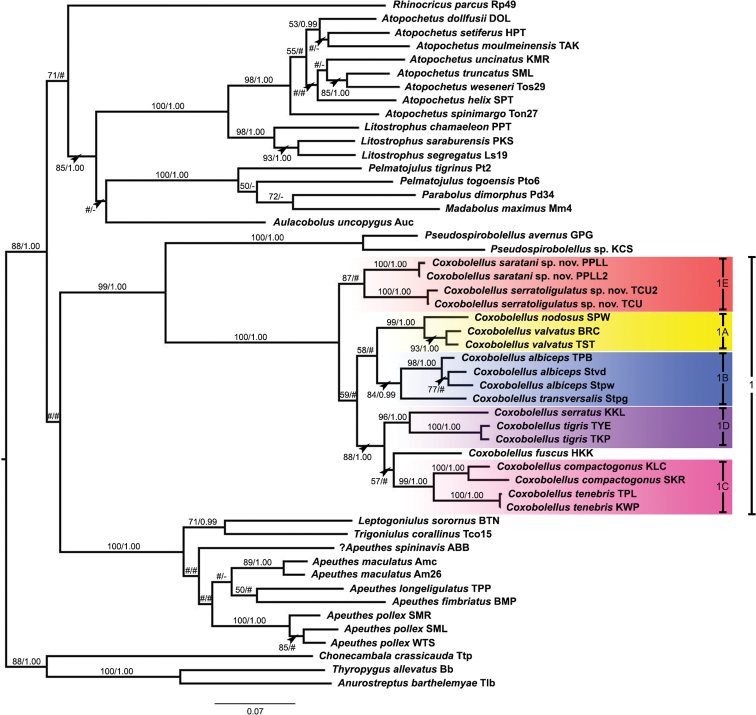
Phylogenetic relationships of *Coxobolellus* species based on maximum likelihood analysis (ML) and Bayesian inference (BI) of 1118 bp in the combined COI + 16S rRNA alignment. Numbers at nodes indicate branch support based on bootstrapping (ML) / posterior probabilities (BI). Scale bar = 0.07 substitutions/site. # indicates branches with <50% ML bootstrap support and <0.95 Bayesian posterior probability. - indicates non-supported branches. The colored areas mark the subclades of *Coxobolellus* and are labelled as in Pimvichai et al. (2020).

The ML and BI trees based on the separate and combined datasets (COI, 16S rRNA and COI + 16S rRNA) were largely congruent with respect to the well-supported branches (by visual inspection of the branching pattern). The combined COI + 16S rRNA tree is used for further discussion (Fig. 1). The separate COI and 16S rRNA trees are presented in Suppl. materials 4, 5, respectively.

The genus *Coxobolellus* (Clade 1) is consistently well supported in the three trees and can be subdivided into five generally well supported subclades (1A–E; cf. Pimvichai et. al. 2020), one of which is formed by the two new species (subclade 1E). Yet, this latter subclade is only well supported by ML of the 16S rRNA (and combined) data, but not by BI and the COI data. Moreover, the position of two other species, viz. *C.fuscus* (COI and 16S rRNA) and *C.simplex* (COI only), remains ambiguous, even if both species seem to be somehow associated with subclades 1C and 1D.

Subclade 1A comprises *C.nodosus* and *C.valvatus*, two species that are distributed in western and northern Thailand, respectively. They differ in their posterior gonopod telopodite: in *C.nodosus* the telopodital part (pt) is as long as the coxal part (pcx), while in *C.valvatus* the telopodital part (pt) is much shorter than the coxal part (pcx). Their COI sequence divergence is 0.06–0.07.

Subclade 1B comprises the sympatric species *C.albiceps* and *C.transversalis*. They mainly differ in the following two aspects: (1) in *C.albiceps* the tip of the anterior gonopod coxa is apically obliquely truncated, while in *C.transversalis* it is transversely truncated; (2) the telopodital part (pt) of the posterior gonopod telopodite is fairly long in *C.transversalis*, while in *C.albiceps* it is short. Their COI sequence divergence is 0.08.

Subclade 1C comprises *C.compactogonus* and *C.tenebris*, two species that are distributed in northeastern and central to western Thailand, respectively. They mainly differ in the following two aspects: (1) the anterior gonopod telopodite (at) ends in a rounded lobe in *C.compactogonus*, while in *C.tenebris* there is a tiny triangular laterad denticle near the tip of the anterior gonpod telopodite (at); (2) the telopodital part (pt) of the posterior gonopod telopodite is with three processes in *C.compactogonus*, while in *C.tenebris* this part ends in a rounded, smooth lobe. Their COI sequence divergence is 0.10 (range: 0.09–0.10).

Subclade 1D comprises *C.serratus* and *C.tigris*, two species that are distributed in southern Thailand. They mainly differ in the following two aspects: (1) in *C.serratus* the posterior gonopod telopodite with a long coxal part (pcx), while this part is short in *C.tigris*; and (2) the telopodital part (pt) laterally is with a serrate margin in *C.serratus*, while in *C.tigris* only the apical part with a serrate margin. Their COI sequence divergence is 0.12.

Subclade 1E (only supported by ML of 16S rRNA) comprises the two new species: *C.saratani* sp. nov. and *C.serratoligulatus* sp. nov., which are distributed in northeastern and northern Thailand, respectively. They differ in both the anterior gonopod and the posterior gonopod telopodite, and will be treated in detail further below. Their COI sequence divergence is 0.08 (range: 0.07–0.08).

The combined COI + 16S rRNA (Fig. 1) and the separate 16S rRNA (Suppl. material 5) trees showed *Pseudospirobolellus* as a well-supported sister group of *Coxobolellus*. However, this sister group relation was no longer supported when the genus *Siliquobolellus* was included, i.e. in the separate COI tree (Suppl. material 4).

### ﻿Taxonomy

#### Class Diplopoda de Blainville in Gervais, 1844

**Order Spirobolida Bollman, 1893**

**Suborder Spirobolidea Bollman, 1893**

##### Family Pseudospirobolellidae Brölemann, 1913

###### ﻿Genus Coxobolellus

Taxon classificationAnimaliaSpirobolidaPseudospirobolellidae

Pimvichai, Enghoff, Panha & Backeljau, 2020

475D37C9-E219-5612-B037-A1A9098A2EB6

####### Diagnosis.

Differing from the other genera of Pseudospirobolellidae by having (1) the coxae of the 3^rd^ pair of male legs with extremely large, protruding processes (in *C.albiceps* and *C.transversalis*, this condition also applies to the 4^th^ pair of male legs), (2) the 4^th^ and 5^th^ leg-pairs with large, triangular coxae, (3) short process of preanal ring protruding as far as, or slightly beyond, anal valves, and (4) the posterior gonopod telopodite divided into a coxal part (pcx) and a telopodital part (pt); with opening of efferent groove (oeg) at mesal margin at the end of coxal part (pcx).

####### Species description.

The two new species share all of the diagnostic characters of the genus *Coxobolellus*, as described in the general description section in Pimvichai et al. (2020: 599–601).

###### ﻿ Coxobolellus saratani sp. nov.

Taxon classificationAnimaliaSpirobolidaPseudospirobolellidae

9430BC29-E8DD-52B2-BFDE-B9077B634973

https://zoobank.org/C8EB9916-1AFE-465E-A630-12970577E4E5

Figs 2 , 4 , 5

####### Material studied.

***Holotype*** ♂ (CUMZ-D00153-1), Thailand, Loei Province, Muang District, Phu Pha Lom; 17°32'30"N, 101°51'38"E; 370 m a.s.l.; 25 September 2021; P. Pimvichai, P. Prasankok and S. Saratan leg. ***Paratypes*.** 7 ♂♂, 9 ♀♀; same data as holotype (CUMZ-D00153-2).

####### Etymology.

The species is named after Mr Sathit Saratan, who always supports the authors during fieldwork and who is a devoted millipede collector.

####### Diagnosis.

**Figure 2.**
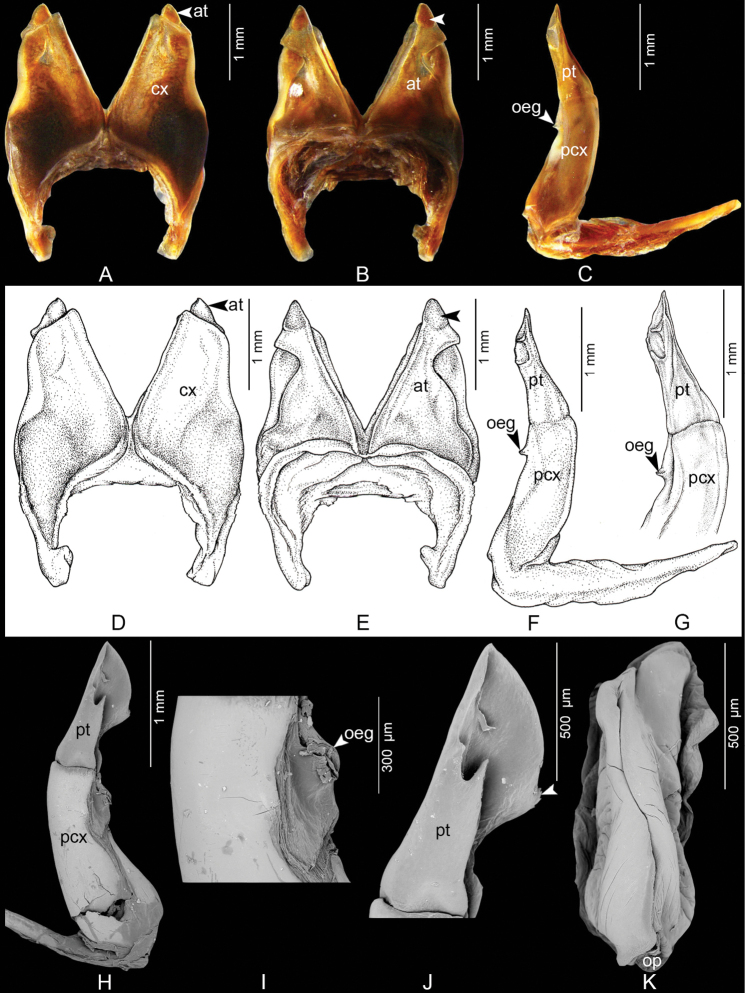
*Coxobolellussaratani* sp. nov., holotype, gonopods (CUMZ-D00153-1) **A, D** anterior gonopod, anterior view **B, E** anterior gonopod, posterior view, unlabeled arrows indicate a pigmented brown node **C, F, G** left posterior gonopod **H**SEM, right posterior gonopod, posterior-mesal view **I**SEM, mesal part of posterior gonopod, posterior-mesal view **J**SEM, tip of posterior gonopod, posterior-lateral view **K**SEM, left female vulva, posterior-mesal view. at = anterior gonopod telopodite; cx = coxa; oeg = opening of efferent groove; op = operculum of vulva; pcx = coxal part of the posterior gonopod telopodite; pt = telopodital part of the posterior gonopod telopodite.

Differing from all other species in the genus by having the tip of the telopodital part (pt) forming a flattened, pointed lobe, directed distad (Fig. 2C, F–H), whereas in the other 11 *Coxobolellus* species the tip of the telopodital part of the posterior gonopod curves mesad or forms a rounded lobe.

####### Description.

Adult males with 51–55 podous rings. Length ca 6–7 cm, diameter ca 4.9–5.2 mm. Adult females with 52 or 53 podous rings. Length ca 6–8 cm, diameter ca 5.6–6.1 mm.

***Colour*.** Living animal greenish grey except for dark brown antennae and legs (Fig. 4A).

***Anterior gonopods*** (Fig. 2A, B, D, E) with high coxae, apically obliquely truncated, mesal margins straight, diverging, delimiting a V-shaped space between both coxae, posterior surface with relatively high ridge laterally for accommodation of telopodite. Telopodite (at) projecting slightly over anterior gonopod coxa (cx), subapically strongly constricted, apically forming a triangular process with pointed tip and a pigmented brown node (Fig. 2B, E, unlabeled arrow).

***Posterior gonopods*** (Fig. 2C, F–J) simple, rounded, with long, smooth coxal part (pcx); telopodital part (pt) fairly long, apically pointed, directed distad, with a sharp, pointed, folded process in the middle (Fig. 2C, F–H), with a small transverse ridge near tip protruding from mesal surface, with serrate mesal margin (Fig. 2J, unlabeled arrow).

***Female vulvae*** (Fig. 2K): valves prominent, of equal size.

####### DNA barcodes.

The GenBank accession numbers are:

Holotype CUMZ-D00153-1: COI = OP580098; 16S rRNA = OP580513.

Paratype CUMZ-D00153: COI = OP580097; 16S rRNA = OP580512.

####### Habitat.

Found under leaf litter and crawling around (on the rock and stairs).

####### Distribution.

Known only from the type locality in Loei Province, Thailand (Fig. 5).

###### ﻿ Coxobolellus serratoligulatus sp. nov.

Taxon classificationAnimaliaSpirobolidaPseudospirobolellidae

20256A6E-838B-59D4-9132-A0AB9AA2AC97

https://zoobank.org/BDE726D9-4EC8-44F8-93D2-AE742C88A793

Figs 3 , 4 , 5

####### Material studied.

***Holotype*** ♂ (CUMZ-D00154-1), Thailand, Uttaradit Province, Thong Saen Khan District, Tham Chan; 17°35'4"N, 100°25'10"E; 230 m a.s.l.; 31 July 2020; P. Pimvichai, P. Prasankok and S. Saratan leg. ***Paratypes*.** 2 ♀♀; same data as holotype (CUMZ-D00154-2).

####### Etymology.

The species epithet is a Latin adjective meaning “with a serrated tongue” and refers to the characteristic process of the posterior gonopod.

####### Diagnosis.

**Figure 3.**
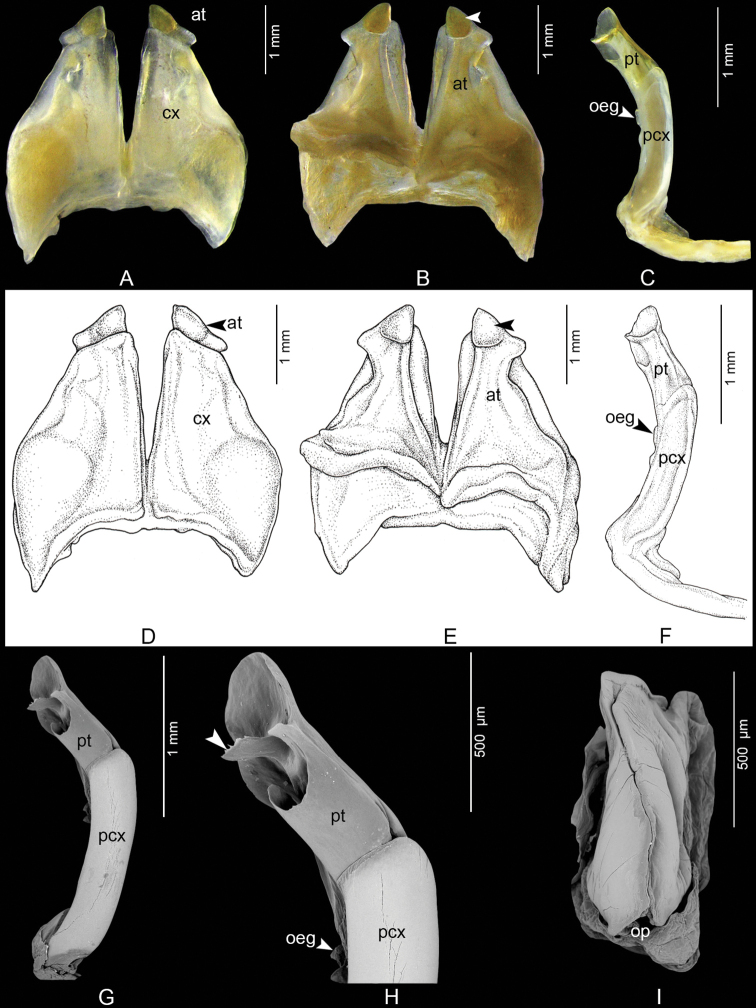
*Coxobolellusserratoligulatus* sp. nov., holotype, gonopods (CUMZ-D00154-1) **A, D** anterior gonopod, anterior view **B, E** anterior gonopod, posterior view, unlabeled arrows indicate a pigmented brown node **C, F** left posterior gonopod **G**SEM, left posterior gonopod, posterior-mesal view **H**SEM, tip of posterior gonopod, posterior-lateral view, unlabeled arrow indicates the tongue-like process **I**SEM, left female vulva, posterior-mesal view. at = anterior gonopod telopodite; cx = coxa; oeg = opening of efferent groove; op = operculum of vulva; pcx = coxal part of the posterior gonopod telopodite; pt = telopodital part of the posterior gonopod telopodite.

Anterior gonopods with high coxae, apically obliquely truncated (Fig. 3A, D). Similar in this respect to *C.albiceps*. Differing from all other species in the genus by having the posterior gonopod with a massive, broad, flattened, serrate process protruding from the mesal surface, forming a tongue-like process (Fig. 3C, F–H), whereas in the other 11 *Coxobolellus* species the telopodital part of the posterior gonopod has no distinct tongue-like process.

####### Description.

Adult male with 54 podous rings. Length ca 5 cm, diameter ca 4.0 mm. Adult females with 51–53 podous rings. Length ca 5 cm, diameter ca 3.9–4.1 mm.

**Figure 4.**
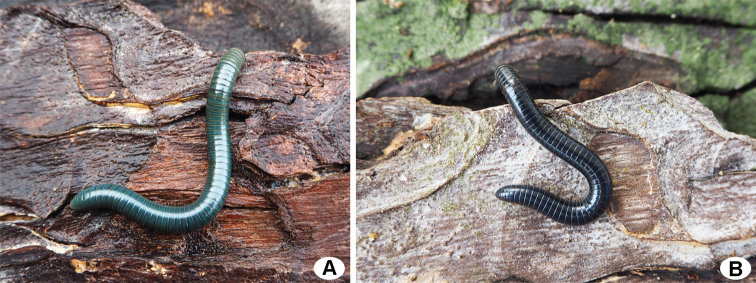
Live *Coxobolellus* species from Thailand **A***C.saratani* sp. nov., male (paratype, CUMZ-D00153-2) **B***C.serratoligulatus* sp. nov., male (holotype, CUMZ-D00154-1).

***Colour*.** Living animal dark green except for dark brown antennae and legs (Fig. 4B).

***Anterior gonopods*** (Fig. 3A, B, D, E) with high coxae, apically obliquely truncated, mesal margins straight, posterior surface with relatively high ridge laterally for accommodation of telopodite. Telopodite (at) projecting over anterior gonopod coxa (cx), subapically strongly constricted, apically forming a big triangular process with pointed tip and a pigmented brown node (Fig. 3B, E, unlabeled arrow).

***Posterior gonopods*** (Fig. 3C, F–H) very simple, rounded, with long, smooth coxal part (pcx); telopodital part (pt) fairly long, curving mesad, ending in a rounded lobe, forming a canopy, with a large, broad, flattened, serrate process protruding from mesal surface, forming a tongue-like process (Fig. 3H, unlabeled arrow).

***Female vulvae*** (Fig. 3I): valves prominent, of equal size.

####### DNA barcodes.

The GenBank accession numbers are:

Holotype CUMZ-D00154-1: COI = OP580100; 16S rRNA = OP580515.

Paratype CUMZ-D00154: COI = OP580099, 16S rRNA = OP580514.

####### Habitat.

Found under leaf litter.

####### Distribution.

**Figure 5.**
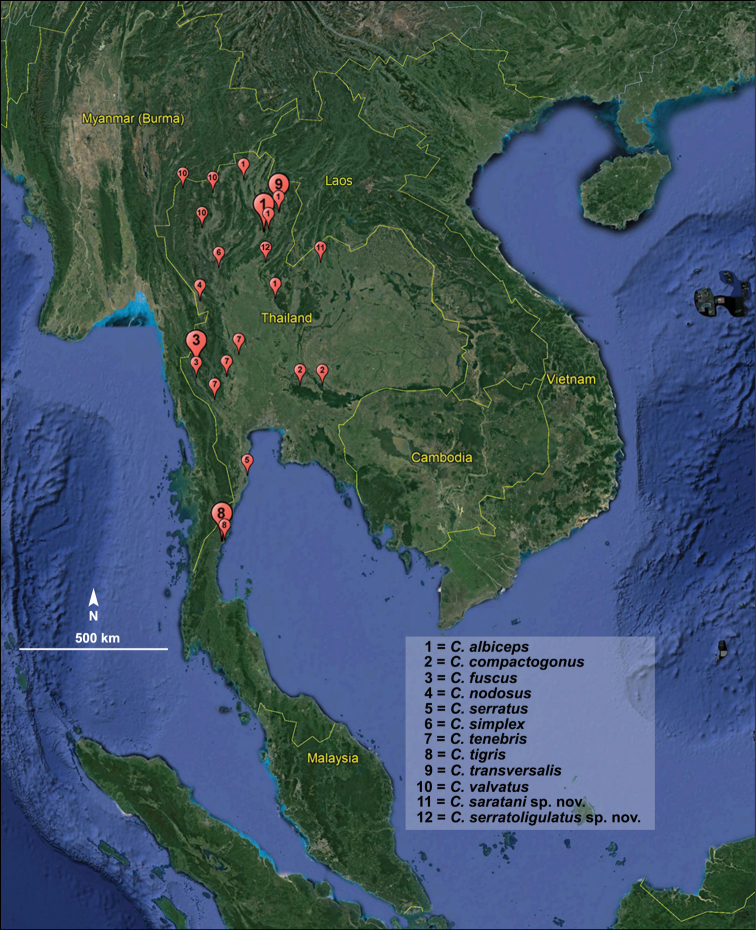
Distribution of the species of *Coxobolellus* in Thailand. Droplets vary in size to improve readability.

Known only from the type locality in Uttaradit Province, Thailand (Fig. 5).

### ﻿Key to species of the genus *Coxobolellus* (based on adult males, update of the key of Pimvichai et al. 2020)

**Table:** 

1	Tip of anterior gonopod coxa truncated	**2**
–	Tip of anterior gonopod coxa concave/bilobed or forming a triangular process	**5**
2	Tip of anterior gonopod coxa transversely truncated; telopodital part (pt) of posterior gonopod long compared to coxal part (pcx)	***C.transversalis* Pimvichai, Enghoff, Panha & Backeljau, 2020**
–	Tip of anterior gonopod coxa obliquely truncated	**3**
3	Telopodital part (pt) of posterior gonopod short compared to coxal part (pcx)	***C.albiceps* Pimvichai, Enghoff, Panha & Backeljau, 2020**
–	Telopodital part (pt) of posterior gonopod fairly long compared to coxal part (pcx)	**4**
4	Telopodital part (pt) directed distad, pointed (Fig. 2C), with a sharp, pointed, folded process in the middle, with a small transverse ridge near tip, with serrate mesal margin (Fig. 2J, unlabeled arrow)	***C.saratani* sp. nov.**
–	Telopodital part (pt) curving mesad, ending in a rounded lobe, forming a canopy, with a broad, flattened, serrate, tongue-like process protruding from mesal surface, (Fig. 3H, unlabeled arrow)	***C.serratoligulatus* sp. nov.**
5	Tip of anterior gonopod coxa concave/bilobed	**6**
–	Tip of anterior gonopod coxa forming triangular process	**7**
6	Tip of anterior gonopod coxa bilobed, outer process broadly rounded, inner process triangular, protruding higher than outer process; telopodital part (pt) of posterior gonopod ending in a rounded margin with a sharp spine protruding from mesal surface near tip	***C.valvatus* Pimvichai, Enghoff, Panha & Backeljau, 2020**
–	Tip of anterior gonopod coxa concave, forming equal outer and inner lobes; telopodital part of posterior gonopod (pt) ending in a long, sharp spine, with a flattened lamella protruding from mesal surface near tip	***C.nodosus* Pimvichai, Enghoff, Panha & Backeljau, 2020**
7	Tip of anterior gonopod coxa ending in an abruptly narrowed, pointed, triangular process	**8**
–	Tip of anterior gonopod coxa ending in a simple triangular process	**10**
8	Tip of anterior gonopod telopodite (at) long, narrow, curving mesad; tip of telopodital part (pt) of posterior gonopod ending in coarsely serrate lamella with a sharp point	***C.fuscus* Pimvichai, Enghoff, Panha & Backeljau, 2020**
–	Tip of anterior gonopod telopodite (at) forming a triangular process	**9**
9	Telopodital part (pt) of posterior gonopod with a sharp, curling lamella at base	***C.tenebris* Pimvichai, Enghoff, Panha & Backeljau, 2020**
–	Telopodital part (pt) of posterior gonopod without a sharp, curling lamella at base	***C.simplex* Pimvichai, Enghoff, Panha & Backeljau, 2020**
10	Anterior gonopod telopodite (at) projecting slightly over anterior gonopod coxa (cx), with rounded tip	***C.compactogonus* Pimvichai, Enghoff, Panha & Backeljau, 2020**
–	Anterior gonopod telopodite (at) far overreaching anterior gonopod coxa (cx), with narrowed tip	**11**
11	Anterior gonopod telopodite (at) directed distad; telopodital part (pt) of posterior gonopod ending in a rounded, serrate margin	***C.tigris* Pimvichai, Enghoff, Panha & Backeljau, 2020**
–	Anterior gonopod telopodite (at) curving laterad; telopodital part (pt) of posterior gonopod laterally with serrate margin	***C.serratus* Pimvichai, Enghoff, Panha & Backeljau, 2020**

## ﻿Discussion

The two new species described here obviously belong to the genus *Coxobolellus* because they share the unique synapomorphic characters of this genus viz., (1) the coxae of the 3^rd^ male leg-pair with extremely large, protruding processes, (2) the 4^th^ and 5^th^ leg-pairs with large, triangular coxae, (3) preanal ring with a short process protruding as far as, or slightly beyond, anal valves, and (4) posterior gonopod telopodite divided into a coxal part (pcx) and a telopodital part (pt); with opening of efferent groove (oeg) at mesal margin at the end of coxal part (pcx) (Pimvichai et al. 2020). In line with this, the mtDNA trees provided maximum support for the placement of the two new species in the monophyletic genus *Coxobolellus*.

The mtDNA data further confirmed the trends observed in earlier studies dealing with levels of COI sequence divergence among millipede species and genera, i.e. high interspecific COI sequence divergences among congeneric species (overall range: 0.05–0.18) (Pimvichai et al. 2014, 2018, 2022a, b; Reip and Wesener 2018), but still clearly higher COI sequence divergences between genera (overall range: 0.16–0.23) (e.g. Pimvichai et al. 2022a, b). Yet, it would be premature to draw taxonomic conclusions from these trends in terms of potential DNA barcode threshold levels.

Similarly, the subdivision of *Coxobolellus* into five subclades should not be interpreted as a prelude to some sort of taxonomic subdivision. Instead, it is an attempt to look for phylogenetic patterns that can help to decide about new species. In the present case, for example, the fact that the two new species tend to form a separate subclade is indeed an additional argument that supports their recognition as new species, even if this subclade is only supported by ML of 16S rRNA.

The inclusion of the two new *Coxobolellus* species in the phylogenetic analysis did not resolve the ambiguous sister group relationships of *Coxobolellus*, *Pseudospirobolellus* and *Siliquobolellus* (Pimvichai et al. 2022b) and neither provided new evidence about the enigmatic position of *Benoitolus*. Obviously, the phylogenetic relationships of the Pseudospirobolellidae need further research.

The present results further reinforce the expectation that new pseudospirobolellid, and in particular *Coxobolellus*, species remain to be discovered in Southeast Asia (cf. Pimvichai et al. 2022b). Although the systematic study of Pseudospirobolellidae started only recently, it has already convincingly shown that this group is far more diverse than hitherto was assumed. The present paper is another testimony of this.

## Supplementary Material

XML Treatment for Coxobolellus

XML Treatment for Coxobolellus saratani

XML Treatment for Coxobolellus serratoligulatus

## Supplementary materials

Supplementary material 1 Estimates of COI sequence divergences within and among *Coxobolellus* species and related taxa expressed as uncorrected p-distances (rounded to two decimals) Data type COI sequence divergences https://binary.pensoft.net/file/767375This dataset is made available under the Open Database License (http://opendatacommons.org/licenses/odbl/1.0/). The Open Database License (ODbL) is a license agreement intended to allow users to freely share, modify, and use this Dataset while maintaining this same freedom for others, provided that the original source and author(s) are credited.Piyatida Pimvichai, Henrik Enghoff, Thierry Backeljau

Supplementary material 2 Estimates of 16S rRNA sequence divergences within and among *Coxobolellus* species and related taxa expressed as uncorrected p-distances (rounded to two decimals) Data type 16S rRNA sequence divergences https://binary.pensoft.net/file/767376This dataset is made available under the Open Database License (http://opendatacommons.org/licenses/odbl/1.0/). The Open Database License (ODbL) is a license agreement intended to allow users to freely share, modify, and use this Dataset while maintaining this same freedom for others, provided that the original source and author(s) are credited.Piyatida Pimvichai, Henrik Enghoff, Thierry Backeljau

Supplementary material 3 Estimates of combine datasets (COI + 16S rRNA) sequence divergences within and among *Coxobolellus* species and related taxa expressed as uncorrected p-distances (rounded to two decimals) Data type COI + 16S rRNA sequence divergences https://binary.pensoft.net/file/767377This dataset is made available under the Open Database License (http://opendatacommons.org/licenses/odbl/1.0/). The Open Database License (ODbL) is a license agreement intended to allow users to freely share, modify, and use this Dataset while maintaining this same freedom for others, provided that the original source and author(s) are credited.Piyatida Pimvichai, Henrik Enghoff, Thierry Backeljau

Supplementary material 4 Phylogenetic relationships of *Coxobolellus* species based on maximum likelihood analysis (ML) and Bayesian inference (BI) of 660 bp of the COI alignment Data type Figure (PDF file) Explanation note Numbers at nodes indicate branch support based on bootstrapping (ML) / posterior probabilities (BI). Scale bar = 0.3 substitutions/site. # indicates branches with <50% ML bootstrap support and <0.95 Bayesian posterior probability. - indicates non-supported branches. https://binary.pensoft.net/file/767378This dataset is made available under the Open Database License (http://opendatacommons.org/licenses/odbl/1.0/). The Open Database License (ODbL) is a license agreement intended to allow users to freely share, modify, and use this Dataset while maintaining this same freedom for others, provided that the original source and author(s) are credited.Piyatida Pimvichai, Henrik Enghoff, Thierry Backeljau

Supplementary material 5 Phylogenetic relationships of *Coxobolellus* species based on maximum likelihood analysis (ML) and Bayesian inference (BI) of 458 bp of the 16S rRNA alignment Data type Figure (PDF file) Explanation note Numbers at nodes indicate branch support based on bootstrapping (ML) / posterior probabilities (BI). Scale bar = 0.06 substitutions/site. # indicates branches with <50% ML bootstrap support and <0.95 Bayesian posterior probability. - indicates non-supported branches. https://binary.pensoft.net/file/767379This dataset is made available under the Open Database License (http://opendatacommons.org/licenses/odbl/1.0/). The Open Database License (ODbL) is a license agreement intended to allow users to freely share, modify, and use this Dataset while maintaining this same freedom for others, provided that the original source and author(s) are credited.Piyatida Pimvichai, Henrik Enghoff, Thierry Backeljau
