# Supplementary material for: ﻿Two new millipede species of the genus Coxobolellus Pimvichai, Enghoff, Panha & Backeljau, 2020 (Diplopoda, Spirobolida, Pseudospirobolellidae)
Source: Zookeys. 2022 Nov 9;1128:171–90. doi: 10.3897/zookeys.1128.94242 (PMC9836734; doi:10.3897/zookeys.1128.94242)
Supplement: Supplementary material 5 — Phylogenetic relationships of Coxobolellus species based on maximum likelihood analysis (ML) and Bayesian inference (BI) of 458 bp of the 16S rRNA alignment [file zookeys-1128-171_article-94242__-s005.pdf]

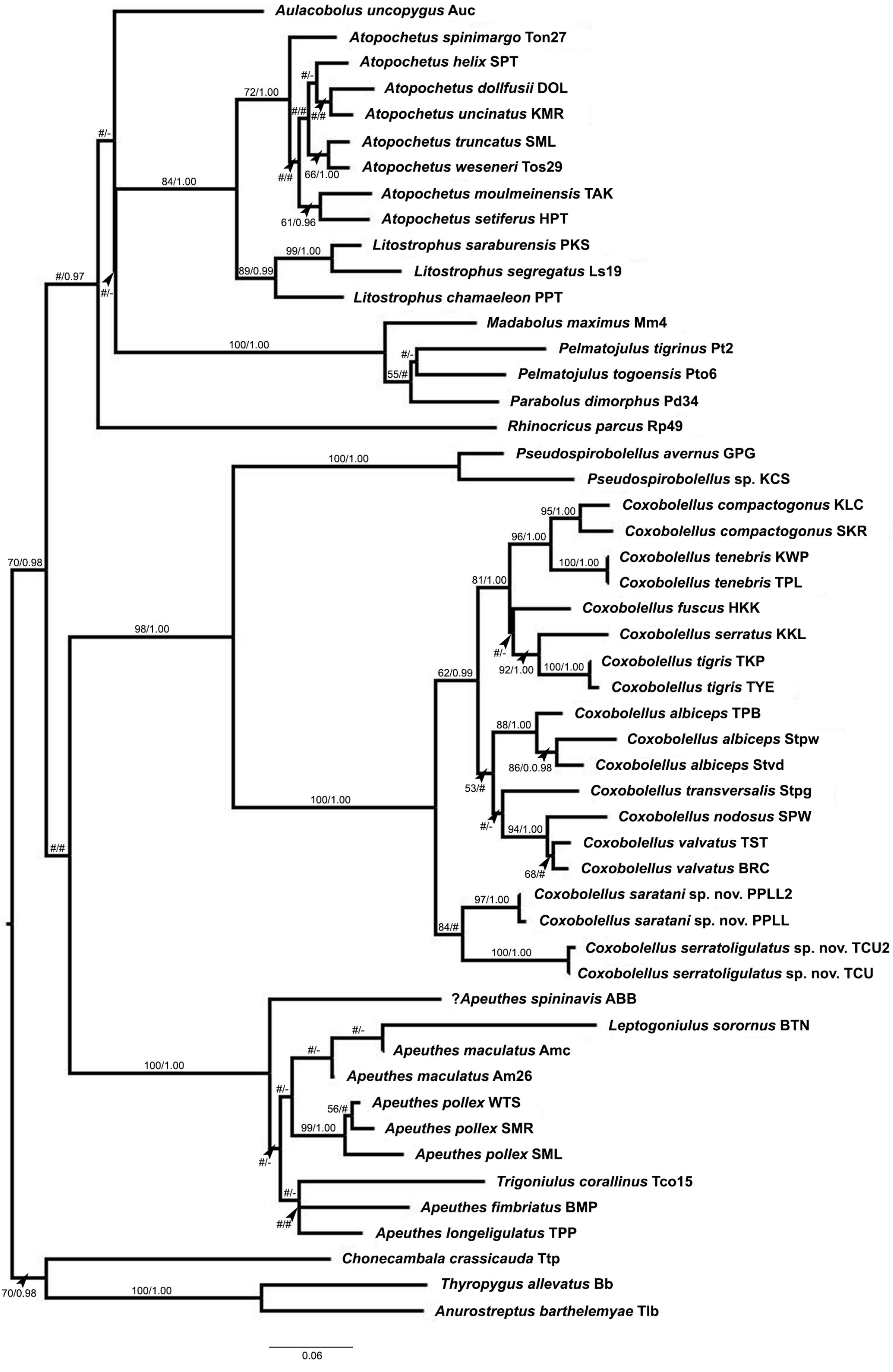

Figure S2. Phylogenetic relationships of *Coxobolellus* species based on maximum likelihood analysis (ML) and Bayesian inference (BI) of 458 bp of the 16S rRNA alignment. Numbers at nodes indicate branch support based on bootstrapping (ML) / posterior probabilities (BI). Scale bar = 0.06 substitutions/site. # indicates branches with <50% ML bootstrap support and <0.95 Bayesian posterior probability. - indicates non-supported branches.
